# Isopropyl Alcohol Intoxication Treated With Hemodialysis: A Case Report and Short Review

**DOI:** 10.7759/cureus.52580

**Published:** 2024-01-19

**Authors:** Sagar Kumar, Sarah Dabbas, FNU Manisha, Huma Akta, Emad Al Jaber

**Affiliations:** 1 Pulmonary Critical Care, University of South Alabama University Hospital, Mobile, USA; 2 Internal Medicine, Springhill Hospital, Mobile, USA; 3 Internal Medicine, Peoples University of Health Sciences, Nawab Shah, PAK; 4 Internal Medicine, Dow University of Health Sciences, Civil Hospital Karachi, Karachi, PAK; 5 Nephrology, University of South Alabama College of Medicine, Mobile, USA

**Keywords:** acute encephalopathy, osmolar gap, severe alcohol intoxication, hemodialysis, isopropyl alcohol intoxication

## Abstract

Isopropyl alcohol (IPA) is a common constituent of rubbing alcohol, household cleaning agents, and antiseptic agents. Ingestion of IPA usually leads to self-resolving mild symptoms in most cases but can result in severe symptoms, including central nervous system depression or hemodynamic instability. Treatment is mainly supportive, and hemodialysis is generally reserved for severe intoxication. Limited data are available on the use of hemodialysis to treat IPA intoxication. We are presenting a case of accidental ingestion of IPA in an elderly female with dementia leading to severe intoxication requiring hemodialysis at relatively non-toxic serum levels of IPA. The patient had a prompt recovery without any post-procedural or hospital-acquired complications.

## Introduction

Alcohol intoxication is a common cause of acute encephalopathy and metabolic imbalances. Common toxicities associated with alcohol in clinical settings include those caused by intoxication with methanol, ethylene glycol, diethylene glycol, and isopropyl alcohol (IPA).

IPA is a clear, colorless liquid with a bitter taste, commonly found in alcohol-based hand solutions and cleaning agents such as rubbing alcohol (usually 70% isopropanol). Intoxication can occur through ingestion or inhalation and dermal exposure in poorly ventilated areas or during alcohol sponge bathing [1]. Typically, IPA intoxication presents mild symptoms, such as nausea and vomiting, or mild central nervous system (CNS) symptoms like headache, visual disturbances, or confusion [2]. In contrast to methanol and ethylene glycol intoxications, which present with an increased osmolar gap with an anion gap metabolic acidosis and usually require hemodialysis, IPA toxicities exhibit a distinctive laboratory pattern of an increased osmolar gap without metabolic acidosis, managed conservatively with supportive measures in the majority of cases [2]. However, in patients with severe intoxication presenting with severe CNS depression, respiratory depression, or severe hypotension, hemodialysis can substantially enhance IPA elimination and may be considered.

We present a case of an elderly patient presenting with severe IPA toxicity characterized by clinical features of severe intoxication, including hemodynamic instability and profound CNS depression, necessitating endotracheal intubation for airway protection. The patient was treated with supportive management and emergent hemodialysis, resulting in rapid recovery, early extubation, and a shortened stay in the intensive care unit.

This case was presented as an abstract in the Spring meeting of the National Kidney Foundation in April 2021 with the abstract title " Dialyze or Not? Case-Based Approach to a Rare Toxicity".

## Case presentation

A 70-year-old African American female with a history of dementia and hypertension was brought to the emergency department (ED) by her son with the chief complaint of unresponsiveness following the accidental ingestion of an entire bottle of rubbing alcohol (isopropyl alcohol). Subsequently, the patient began vomiting gastric contents, and her mental status declined to a completely unresponsive state with a Glasgow Coma Scale (GCS) of 3, necessitating intubation for airway protection. There was no history of trauma, prior suicide attempts, or psychiatric illness. Her medication regimen included amlodipine, carvedilol, atorvastatin, clonidine, meloxicam, pantoprazole, and potassium chloride. She had no known drug allergies.

The physical examination revealed a blood pressure of 91/52 mmHg, a pulse of 83 beats per minute, a temperature of 36.4 degrees Celsius, and a respiratory rate of 6 breaths per minute. The patient appeared somnolent and obtunded. The sclerae were anicteric, conjunctivae were injected, and pupils were 4 mm in diameter and reactive. Heart and chest examinations were unremarkable. The neurological examination did not reveal any other focal abnormalities, and no skin rash was noted. The patient required 2 liters of normal saline boluses and a phenylephrine injection in the ED, resulting in an adequate response.

Her basic laboratory workup results upon presentation, including toxicology workup, are presented in Table 1. Imaging, including a contrast-free computerized tomography (CT) head scan, revealed no significant changes except for a small chronic right frontal infarct.

**Table 1:** Baseline laboratory values and toxicology results mg/dL - Milligrams per deciliter, mMol/L - Millimoles per liter, mcL - Microliter, g/dL - Grams per deciliter, mOsm/kg - Milliosmoles per kilogram BUN - Blood Urea Nitrogen, CO2 - Bicarbonate, WBC - White Blood Cells, MDMA - 3,4-Methylenedioxymethamphetamine,NA - Not applicable

Lab Name	Lab Value	Reference Range
Serum glucose	162 mg/dL	70-110 mg/dl
BUN	23 mg/dL	7-18mg/dl
Creatinine	1.53 mg/dL	0.53-1.02 mg/dl
Sodium	145 mMol/L	136-145 mMol/L
Potassium	4.1 mMol/L	3.5-5.1 mMol/L
Chloride	114 mMol/L	98- 107 mMol/L
CO2	22 mMol/L	22-28 mMol
Lactate	1.9 mMol/L	<2 mMol/L
WBC	12.92 x10(3)/mcL	4.0 x10(3) – 11.0x10(3)/mcL
Hemoglobin	13.4 g/dL	12.0- 15.0 g/dl
Platelet count	242 x10(3)/mcL	150-450 x 10(3)/mcL
Serum osmolality	359 mOsm/kg	275 to 295 mOsm/kg.
Plasma acetone	Small	Undetectable
Ethylene glycol	<5 mg/dL	<10 mg/dL
Ethanol	<3 mg/dL	<10 mg/dL
Methanol level	<5 mg/dL	Detectable at >5mg/dl
Isopropyl alcohol level	82 mg/dL	<10 mg/dL
Urine amphetamine screen	Not Detected	NA
Urine benzodiazepine screen	Not Detected	NA
Urine cocaine screen	Not Detected	NA
Urine opiate screen	Not Detected	NA
Urine MDMA screen	Not Detected	NA

The patient was admitted to the intensive care unit, and the nephrology team was consulted for assistance with the patient’s management. Fomepizole was not administered, as it can worsen IPA intoxication symptoms. The decision was made to perform hemodialysis due to altered mental status, respiratory depression, and relative hypotension. The patient underwent hemodialysis for IPA alcohol intoxication. The patient was reassessed within a few hours after the hemodialysis session, and her mentation had significantly improved, resulting in extubation within a few hours.

The patient remained stable throughout further hospitalization and was discharged home on her prescribed antihypertensives.

## Discussion

IPA is approximately twice as potent as ethanol in causing CNS depression, and its duration of action is two to four times that of ethanol [2]. Accidental ingestion is common in children [3], and cases of intentional and unintentional severe intoxications are reported in adults, especially those with alcohol use disorder [2]. IPA is rapidly absorbed following ingestion, with mammalian studies indicating that 99% of an orally administered dose is absorbed within 2 hours, and peak plasma concentration occurs within 30 minutes. After ingestion, IPA breaks down into isopropanol and acetone, primarily excreted through the kidneys and minimally through the lungs [4]. Most cases of IPA intoxication are self-resolved without major clinical consequences [3,5,6]. Mortality is minimal, reported as 0.1% in 2004 [6], and there have been no reported deaths in more recent data after that despite tens of thousands of cases [5].

Clinical features

The minimal dose of IPA resulting in acute symptoms has not been established. Adults administered 20-30 mL of a 50% solution of IPA developed only mild symptoms and signs [4]. The exact mechanism of IPA toxicity remains not fully understood, but peripheral vasodilation or decreased cardiac inotropy may contribute to hypotension. It is a sedative agent whose toxicity closely resembles that of ethanol, with a strong structural similarity [4].

The most common feature observed is CNS depression, with symptoms ranging from lethargy or drowsiness to stupor and coma. Other common neurological effects include hyperreflexia, hypotonia, ataxia, and headache [1]. Gastrointestinal distress, including nausea, vomiting, or hematemesis secondary to hemorrhagic gastritis [1,2], are also common early symptoms.

Additional clinical features encompass respiratory depression, hypotension, circulatory failure, renal failure, or pseudo-renal failure due to acetone interference [4,7,8]. In addition to acute intoxication, chronic neuromuscular toxicities, including cerebellar dysfunction, dementia, rhabdomyolysis, and myopathy, have been reported with prolonged exposure to IPA [9]. Hypotension and coma indicate severe intoxication and require prompt interventions that may include hemodialysis [10-13]. Isopropanol is the primary toxic compound causing most of these symptoms [10,12,14]. Previously, acetone was thought to be responsible for CNS depression following IPA exposure; however, recent case reports suggest that isopropanol itself is the major contributor to CNS depression, as clinical improvement has been observed while acetone concentrations were still rising [6].

Laboratory findings in isopropyl alcohol intoxication include ketonemia, ketonuria, an increased osmolar gap without metabolic acidosis, and elevated creatinine due to renal failure. A fruity or sweet odor on the breath may occur [7]. Isopropanol is metabolized to a ketone, not an acid; therefore, ketosis and an osmolar gap without metabolic acidosis are the hallmarks of isopropanol intoxication.

Metabolic acidosis in such patients usually indicates co-ingestion of other toxins and is crucial to identify, as early intervention with fomepizole, ethanol, or hemodialysis can significantly improve morbidity and mortality [15,16]. Additionally, lactic acidosis secondary to hypoperfusion and circulatory collapse can contribute to metabolic acidosis [10]. On the contrary, increased levels of isopropanol are reported in postmortem analysis of patients with diabetic ketoacidosis and Alcoholic ketoacidosis due to the endogenous conversion of acetone to isopropanol [17,18].

Treatment

The management of IPA intoxication is generally supportive, given the major complications involving CNS depression, circulatory failure, and respiratory depression. Close monitoring for respiratory and cardiovascular compromise is essential. Stabilization includes appropriate airway management, including intubation and ventilation for obtunded patients, securing IV access, administration of IV fluids, and cardiac monitoring, with a preference for critical care units equipped with central hemodynamic monitoring. Poor prognostic factors encompass hypotension, severe lactic acidosis, and serum isopropanol levels exceeding 200-400 mg/dL [10].

Decontamination methods, such as gastric lavage and activated charcoal, lack proven benefits due to rapid IPA absorption into the system or the requirement of unusually high amounts of charcoal. The risks of these decontamination methods likely outweigh the potential benefits [4].

Fomepizole, an alcohol dehydrogenase inhibitor, should be avoided in IPA intoxication, as it may worsen symptoms by inhibiting the conversion of isopropanol to acetone. Since the toxicity of isopropanol primarily stems from the parent alcohol, inhibiting its metabolism could lead to prolonged CNS depression, hypotension, or respiratory depression.

Hemodialysis emerges as an effective method for toxin removal in IPA intoxication. Isopropanol and acetone, with their low molecular weight, relatively low volumes of distribution, and minimal serum protein binding, are amenable to removal by extracorporeal techniques like hemodialysis and hemodiafiltration. Rosinsky et al. reported hemodialysis to be 52 times more effective in removing isopropanol compared to urinary excretion [19]. Hemodialysis is generally reserved for severe cases involving coma, refractory hypotension, respiratory depression, renal failure, or extremely high serum IPA levels [11,12,20].

## Conclusions

The treatment of IPA intoxication is primarily supportive, with hemodialysis indicated in the presence of coma, respiratory depression, or marked hypotension. This case contributes to the literature on severe isopropyl alcohol intoxication, highlighting the significance of timely hemodialysis in cases leading to CNS and respiratory depression. Hemodialysis resulted in rapid recovery, shortened overall intubation duration, and reduced intensive care unit stay in an elderly patient. An intriguing aspect is that our patient developed severe symptoms at relatively non-toxic levels (82 mg/dL) compared to the typically reported toxic levels of 200-400 mg/dL in various literature. This could have been secondary to multiple risk factors for CNS depression present in our patient, including a history of strokes, dementia, age, or a combination of all these factors.
